# History, Physical Examination, and Laboratory Findings Associated with Infection and the Empiric Treatment of Gonorrhea and Chlamydia of Women in the Emergency Department

**DOI:** 10.7759/cureus.6482

**Published:** 2019-12-27

**Authors:** Johnathan M Sheele, Justin Smith, Joshua D Niforatos, Emily Wessling, Benjamin Hilliker, Bradley Bragg, Ed Mandac

**Affiliations:** 1 Emergency Medicine, Mayo Clinic, Jacksonville, USA; 2 Surgery, Division of Vascular Surgery and Endovascular Therapy, Case Western Reserve University, Cleveland, USA; 3 Emergency Medicine, Cleveland Clinic Lerner College of Medicine, Case Western Reserve University, Cleveland, USA; 4 Emergency Medicine, Northwestern Memorial Hospital, Chicago, USA; 5 Emergency Medicine, University Hospitals Cleveland Medical Center, Cleveland, USA

**Keywords:** neisseria gonorrhea, chlamydia trachomatis, sexually transmitted infection, emergency medicine, treatment, antibiotic, emergency department

## Abstract

Background

Neisseria gonorrhea (NG), Chlamydia trachomatis (CT), and Trichomonas vaginalis (TV) are common sexually transmitted infections (STIs) treated in the emergency department (ED).

Objectives

To assess the history, physical examination, and laboratory findings associated with NG and CT infection and the decision to administer empiric antibiotic treatment for the diseases in the ED.

Methods

A retrospective review of 566 clinical encounters of adult female patients tested for STIs between January 1, 2013 and December 31, 2014. An analysis of patient- and provider-level variables was assessed to determine the characteristics associated with empiric antibiotic treatment in the ED and post-discharge laboratory confirmed NG or CT.

Results

Younger age and the presence of TV on vaginal wet prep had a higher association with being infected with NG or CT (p < 0.05). Subjective exam findings, such as vaginal discharge, abdominal pain, urinary urgency, urinary frequency, dysuria, objective vaginal discharge, cervical motion tenderness, adnexal tenderness, vaginal bleeding, as well as positive leukocyte esterase and nitrites on urinalysis were all not associated with NG or CT infection (p > 0.05). ED providers were more likely to treat subjects in the ED for NG and CT when there was subjective and objective vaginal discharge, cervical motion tenderness, adnexal tenderness, and vaginal bleeding, TV on wet prep, and leukocyte esterase on urinalysis (p < 0.05).

Conclusions

Only younger age women and the presence of TV on vaginal wet prep were associated with NG or CT infection. ED providers empirically over-treated with antibiotics ~20 patients uninfected with NG and CT by laboratory confirmation, for every one patient with a laboratory confirmed infection.

## Introduction

*Neisseria gonorrhea* (NG) and *Chlamydia trachomatis* (CT) are common bacterial sexually transmitted infections (STIs) and are leading causes of pelvic inflammatory disease, infertility, and ectopic pregnancy in the United States [1-2]. STIs are frequently encountered in the emergency department (ED) with the incidence rising ~39% between 2008-2013 [3]. Current recommendations for diagnosing NG and CT in the ED utilize nucleic acid amplification testing (NAAT) [4]. Since the results of the NAAT are not available to clinicians at the time of the clinical encounter, they must rely on their history and physician examination as well as any ancillary laboratory findings to determine whether empiric antibiotics for NG and CT should be administered, or treatment deferred until the NAAT results are available [5].

*N. gonorrhea* and CT infections can range from asymptomatic to severe systemic illness, with women generally presenting with mucopurulent vaginal discharge, urinary complaints, and pain. The symptoms and urinalysis findings of NG and CT can overlap with cystitis [6-7]. The lack of a reliable rapid diagnostic test for NG and CT can result in an overtreatment of patients without a sexually transmitted infection (STI) as well as misdiagnose patients with genital-urinary complaints [5-7]. The objectives of our study were to determine the history, physical examination, and laboratory findings associated with NG and CT infection in women and determine which variables are associated with empiric antibiotic treatment for STI in the ED.

## Materials and methods

A retrospective study from a single academic tertiary care emergency department in Cleveland, Ohio was conducted between January 1, 2013 and December 31, 2014. Patients were included if they were female, ≥18 years of age, and tested for NG and CT by the APTIMA NAAT (Hologic, Marlborough, MA) or for Trichomonas vaginalis (TV) by the APTIMA NAAT or vaginal wet prep microscopy. Patients with stable vital signs, a triage emergency severity index (ESI) level 4-5, and who presented to the ED between 9 am-5 pm on Monday-Friday could be triaged at the point of clinical contact to a medical access clinic in which their clinical encounter would not be included in our dataset. There were 7,475 encounters that met inclusion criteria and from which a random convenience sample of 566 (8%) patient charts were manually reviewed. Data extracted from each patient’s chart included the age, provider documentation of history and physical exam findings, laboratory results, and whether an antibiotic for STI was given in the ED. Antibiotics for NG/CT included ceftriaxone plus azithromycin or doxycycline. Subjects were considered infected or uninfected with NG or CT if they tested positive or negative by NAAT, respectively. Because the sensitivity of the vaginal wet prep ranges from only 51-65% for TV, if a subject had a negative wet prep the clinician could then order a NAAT [5]. Categorical variables were summarized by frequency or percentage and analyzed using Chi-square. An alpha of 0.05 was set for statistical significance. This study was approved by the University Hospitals (UH) Institutional Review Board.

## Results

Of the 566 charts reviewed, 12% of patients (n = 68) were under the age of 30 years and 39% were over the age of 40 years (n = 223). However, 10% (7/68) of persons under the age of 30 years were infected with NG and/or CT compared to 0.9% (2/221) of subjects over the age of 40 years. The number of patients over the age of 30 that were infected with NG or CT (2% [10/481]) was significantly less than those under the age of 30 (p < 0.001). There was only one subject (0.2%) co-infected with both NG and CT, and 532 (94%) subjects that were not infected with either NG or CT.

Of the 526 patients with wet prep microscopy ordered at the ED visit, 12% (n = 64) of patients had confirmed TV, and 10% (34/353) were positive for TV by NAAT. Seven percent (n = 22/328) of patients with a negative wet prep for TV were positive for TV by NAAT. Those patients infected with TV by wet prep or NAAT were significantly more likely to be co-infected with either NG or CT (8% [7/89]) than those uninfected with TV (2.3% [10/442]) (p < 0.006). Overall, 83% (442/531) of patients were not infected with NG, CT, or TV (by NAAT or wet prep).

The only variables significantly associated with NG or CT infection in the ED were a younger age and those patients positive for TV on vaginal wet prep (p < 0.05) (Tables 1-2).

**Table 1:** Age and subjective complaints associated with infection with gonorrhea or chlamydia in the emergency department.

	+gonorrhea or chlamydia	-gonorrhea or chlamydia	p-value
Age	<30: 41% (7/17) ≥ 30: 59% (10/17)	<30: 12% (61/532) ≥ 30: 89% (471/532)	<0.001
Reported vaginal discharge	Yes: 41% (7/17) No: 59% (10/17)	Yes: 32% (167/526) No: 68% (359/526)	0.41
Reported abdominal pain	Yes: 53% (9/17) No: 47% (8/17)	Yes: 58% (309/529) No: 42% (220/529)	0.65
Reported urinary urgency	Yes: 0% (0/16) No: 100% (16/16)	Yes: 4% (23/517) No: 96% (494/517)	0.39
Reported urinary frequency	Yes: 0% (0/16) No: 100% (16/16)	Yes: 10% (52/516) No: 90% (464/516)	0.18
Reported dysuria	Yes: 6% (1/16) No: 94% (15/16)	Yes: 12% (64/520) No: 88% (456/520)	0.46

**Table 2:** Physical examination and laboratory findings associated with infection with gonorrhea or chlamydia in the emergency department. ED: Emergency department

	+gonorrhea or chlamydia	-gonorrhea or chlamydia	p-value
Vaginal discharge noted on pelvic exam	Yes: 57% (8/14) No: 43% (6/14)	Yes: 51% (244/478) No: 49% (234/478)	0.65
Cervical motion tenderness noted on pelvic exam	Yes: 7% (1/14) No: 93% (13/14)	Yes: 10% (47/475) No: 90% (428/475)	0.73
Adnexal tenderness noted on pelvic exam	Yes: 23% (3/13) No: 77% (10/13)	Yes: 13% (61/466) No: 87% (405/466)	0.30
Vaginal bleeding noted on pelvic exam	Yes: 38% (5/13) No: 62% (8/13)	Yes: 23% (112/481) No: 77% (369/481)	0.20
Diagnosed clinically with a urinary tract infection in the ED	Yes: 19% (3/16) No: 81% (13/16)	Yes: 13% (71/526) No: 87% (455/526)	0.55
Trichomonas vaginalis found on vaginal wet prep	Yes: 47% (7/15) No: 53% (8/15)	Yes: 11% (57/502) No: 89% (445/502)	<0.001
Leukocyte esterase present on urinalysis	Yes: 57% (8/14) No: 43% (6/14)	Yes: 70% (334/479) No: 30% (145/479)	0.31
Urine nitrites present on urinalysis	Yes: 7% (1/14) No: 93% (13/14)	Yes: 3% (16/478) No: 97% (462/478)	0.44

Subjective vaginal discharge, abdominal pain, urinary urgency, urinary frequency, dysuria, as well as the presence of vaginal discharge, cervical motion tenderness, adnexal tenderness, or vaginal bleeding on pelvic exam were not associated with NG or CT infection (p > 0.05) (Tables 1-2). The presence of leukocyte esterase and urine nitrites on urinalysis were also not associated with NG or CT infection (p > 0.05) (Table 2).

ED providers were significantly more likely to treat ED patients for NG and CT when the following variables were present in univariate analysis: subjective and objective vaginal discharge, cervical motion tenderness, adnexal tenderness, vaginal bleeding on pelvic exam, the presence of TV on wet prep, and leukocyte esterase on urinalysis (p < 0.05) (Table 3).

**Table 3:** History, physical examination, and laboratory findings associated with the treatment for gonorrhea and chlamydia in the emergency department. ED: Emergency department

	Treated for gonorrhea or chlamydia in the ED	Not treated for gonorrhea or chlamydia in the ED	p-value
Age	<30: 9% (8/88) ≥ 30: 91% (80/88)	<30: 13% (63/474) ≥ 30: 87% (411/474)	0.28
Reported vaginal discharge	Yes: 51% (44/87) No: 49% (43/87)	Yes: 29% (136/467) No: 71% (331/467)	<0.001
Reported abdominal pain	Yes: 61% (54/88) No: 39% (34/88)	Yes: 58% (272/471) No: 42% (199/471)	0.53
Reported urinary urgency	Yes: 2% (2/86) No: 98% (84/86)	Yes: 5% (24/459) No: 95% (435/459)	0.25
Reported urinary frequency	Yes: 9% (8/86) No: 91% (78/86)	Yes: 11% (49/458) No: 89% (409/458)	0.70
Reported dysuria	Yes: 19% (16/86) No: 81% (70/86)	Yes: 11% (53/462) No: 89% (409/462)	0.07
Vaginal discharge noted on pelvic exam	Yes: 78% (68/87) No: 22% (19/87)	Yes: 46% (189/413) No: 54% (224/413)	<0.001
Cervical motion tenderness noted on pelvic exam	Yes: 28% (24/86) No: 72% (62/86)	Yes: 6% (24/410) No: 94% (386/410)	<0.001
Adnexal tenderness noted on pelvic exam	Yes: 23% (19/82) No: 77% (63/82)	Yes: 11% (46/404) No: 88% (358/404)	0.004
Vaginal bleeding noted on pelvic exam	Yes: 11% (10/87) No: 89% (77/87)	Yes: 26% (110/416) No: 74% (306/416)	0.003
Diagnosed clinically with a urinary tract infection in the ED	Yes: 17% (15/88) No: 83% (73/88)	Yes: 14% (64/467) No: 86% (403/467)	0.41
Trichomonas vaginalis found on vaginal wet prep	Yes: 29% (25/86) No: 71% (61/86)	Yes: 9% (38/438) No: 91% (400/438)	<0.001
Leukocyte esterase present on urinalysis	Yes: 58% (46/80) No: 43% (34/80)	Yes: 71% (300/424) No: 29% (124/424)	0.02
Urine nitrites present on urinalysis	Yes: 4% (3/79) No: 96% (76/79)	Yes: 3% (14/424) No: 97% (410/424)	0.82

Overall, 16% of patients (85/547) that were tested for both NG and CT in the ED were also empirically treated for the disease. ED providers correctly treated 24% (4/17) of patients infected with NG or CT with antibiotics in the ED; however, these providers also empirically treated with antibiotics 15% (81/530) of patients with neither NG nor CT infection (Table 4).

**Table 4:** List of patient characteristics associated with gonorrhea or chlamydia infection and whether the patient was treated for the disease. ED: Emergency department; NG: Neisseria gonorrhoeae; CT: Chlamydia trachomatis.

	+NG/CT and +Rx	+NG/CT and -Rx	-NG/CT and +Rx	-NG/CT and -Rx
Age	<30 years: 3% (2/67) ≥30 years: 0.5% (2/480)	<30 years: 8% (5/67) ≥30 years: 2% (8/480)	<30 years: 8% (5/67) ≥30 years: 16% (76/480)	<30 years: 82% (55/67) ≥30 years: 82% (394/480)
Reported vaginal discharge	Yes: 1% (2/174) No: 0.5% (2/367)	Yes: 3% (5/174) No: 2% (8/367)	Yes: 22% (39/174) No: 11% (41/367)	Yes: 74% (128/174) No: 86% (316/367)
Reported abdominal pain	Yes: 1% (2/317) No: 1% (2/227)	Yes: 2% (7/317) No: 3% (6/227)	Yes: 16% (50/317) No: 14% (31/227)	Yes: 81% (258/317) No: 83% (188/227)
Reported urinary urgency	Yes: 0% (0/23) No: 1% (4/508)	Yes: 0% (0/23) No: 2% (12/508)	Yes: 9% (2/23) No: 15% (77/508)	Yes: 91% (21/23) No: 82% (415/508)
Reported urinary frequency	Yes: 0% (0/52) No: 1% (4/478)	Yes: 0% (0/52) No: 3% (12/478)	Yes: 15% (8/52) No: 15% (71/478)	Yes: 85% (44/52) No: 82% (391/478)
Reported dysuria	Yes: 0% (0/65) No: 1% (4/469)	Yes: 2% (1/65) No: 2% (11/469)	Yes: 23% (15/65) No:14% (64/469)	Yes: 75% (49/65) No: 83% (390/469)
Vaginal discharge noted on pelvic exam	Yes: 2% (4/251) No: 0% (0/240)	Yes: 2% (4/251) No: 3% (6/240)	Yes: 25% (62/251) No: 8% (18 (240)	Yes: 72% (181/251) No: 90% (216/240)
Cervical motion tenderness noted on pelvic exam	Yes: 0% (0/47) No: 1% (3/441)	Yes: 2% (1/47) No: 2% (10/441)	Yes: 49% (23/47) No: 13% (57/441)	Yes: 49% (23/47) No: 84% (371/441)
Adnexal tenderness noted on pelvic exam	Yes: 0% (0/63) No: 1% (3/415)	Yes: 5% (3/63) No: 2% (7/415)	Yes: 29% (18/63) No: 14% (58/415)	Yes: 67% (42/63) No: 84% (347/415)
Vaginal bleeding noted on pelvic exam	Yes: 0% (0/117) No: 1% (4/376)	Yes: 4% (5/117) No: 1% (4/376)	Yes: 9% (10/117) No: 19% (70/376)	Yes: 87% (102/117) No: 79% (298/376)
Diagnosed clinically with a urinary tract infection in the ED	Yes: 1% (1/74) No: 1% (3/467)	Yes: 3% (2/74) No: 2% (10/467)	Yes: 19% (14/74) No: 14% (67/467)	Yes: 77% (57/74) No: 83% (387/467)
Trichomonas vaginalis found on vaginal wet prep	Yes: 5% (3/63) No: 0.2% (1/452)	Yes: 6% (4/63) No: 2% (7/452)	Yes: 35% (22/63) No: 13% (57/452)	Yes: 54% (34/63) No: 86% (387/452)
Leukocyte esterase present on urinalysis	Yes: 2% (3/150) No: 0.3% (1/341)	Yes: 2% (3/150) No: 2% (7/341)	Yes: 19% (29/150) No: 13% (44/341)	Yes: 77% (115/150) No: 85% (289/341)
Urine nitrites present on urinalysis	Yes: 0% (0/17) No: 1% (4/473)	Yes: 6% (1/17) No: 2% (9/473)	Yes: 18% (3/17) No: 15% (69/473)	Yes: 77% (13/17) No: 83% (391/473)

Since there were many more uninfected patients in our dataset, ED providers empirically over-treated with antibiotics ~20 patients uninfected with NG and CT by laboratory confirmation, for every one patient with a laboratory confirmed infection. The sensitivity and specificity of ED providers empirically treating NG and/or CT in the ED was 24% (95% CI 7-50%) and 85% (95% CI 81-88%), respectively.

## Discussion

The signs and symptoms of infection with NG and CT can be nonspecific or absent. Recently the utility of performing pelvic exams in the ED has been questioned as it may not provide additional helpful information for predicting NG and CT infection outside of what is obtained from the patient history [8]. Our results suggest that finding on pelvic exam could bias providers towards treating for NG and CT when the infection is not present.

On univariate analysis there were multiple variables associated with ED providers empirically treating patients with antibiotics for NG and CT in the ED; however, none of these variables were associated with an increased risk for infection with NG or CT with the exception of TV on vaginal wet prep. Other studies have shown additional associated risk factors for STIs in the ED including male sex, women <25 years of age, being diagnosed with pelvic inflammatory disease, penile discharge, and patients without health insurance [9]. Clinicians significantly over-treated NG and CT in the ED. The preliminary results presented here could help in the development of a clinical decision rule to aid providers’ clinical judgement on the administration of empiric antibiotics specifically for NG and CT. This could potentially improve antibiotic stewardship by reducing inappropriate over-prescribing of antibiotics when the infection is not present. Antibiotics may still be warranted for some genital tract infections such as pelvic inflammatory disease where NG and CT are present in <50% of cases [4]. Considering the pretest probability for an STI may affect the decision to empirically treat patients, as STI incidence rates of patient populations have demonstrated significant variation in hospital-based EDs (22%) versus free-standing EDs (9%) [9].

Limitations

The study was retrospective and from a single center, and practice patterns of clinicians at this institution may not be representative of those across the country. Some low-risk patients presenting to the ED were triaged to a medical access clinic and not able to be included in the dataset which could have skewed the data. Furthermore, this study was limited by what was charted. Clinicians may not have documented all the history and physical exam findings in the medical record, regardless of what was actually said and seen during the encounter. Therefore, history and physical exam findings were only helpful if they were clearly recorded. The analyzed data was abstracted from 8% of all available clinical encounters that met criteria (566/7,475) and this could affect generalizability of the results. Only 3.1% (17/566) of subjects tested positive for NG or CT.

## Conclusions

Most of the history, physical exam, and laboratory variables examined in this study were not associated with NG or CT infection. In this analysis, only younger age and the presence of TV on vaginal wet preparation showed a significant association with NG or CT infection. Subjective and objective vaginal discharge, cervical motion tenderness, adnexal tenderness, and vaginal bleeding on pelvic exam were all significantly associated with clinicians treating the patient in the ED with antibiotics for NG and CT infection. However, most patients treated in the ED with antibiotics for NG and CT did not actually have one of the diseases.
